# Zero Risk Is Impossible: Confidentiality and Artificial Intelligence Use in Peer Review

**DOI:** 10.31662/jmaj.2026-0060

**Published:** 2026-06-05

**Authors:** Shigeki Matsubara

**Affiliations:** 1 Department of Obstetrics and Gynecology, Jichi Medical University, Tochigi, Japan; 2 Department of Obstetrics and Gynecology, Koga Red Cross Hospital, Koga, Japan; 3 Medical Examination Center, Ibaraki Western Medical Center, Chikusei, Japan

**Keywords:** artificial intelligence, ChatGPT, confidentiality, peer review, review

## Abstract

Most journals’ and publishers’ guidelines regarding the use of artificial intelligence (AI) in peer review contain a similar statement: “reviewers are prohibited from uploading the manuscript to software or AI-assisted tools or technologies where confidentiality is not assured.” I believe that reviewers should refrain from uploading not only the submitted manuscript but also their own review sheets to AI tools. This position is based on two reasons. First, even if AI tools state that uploaded data will be deleted upon request, actual deletion cannot be independently confirmed. Second, reviewers sometimes include highly confidential keywords or messages in the review sheet; uploading them may jeopardize confidentiality. My humble experiment indicates that even when deletion of uploaded data is requested, AI systems such as ChatGPT may retain materials for training purposes; thus, complete deletion is theoretically impossible. Authors are usually permitted to upload their own manuscripts for linguistic refinement. Although this may also jeopardize confidentiality, authors do so voluntarily and accept that risk. Reviewers, however, are entrusted with unpublished work and should therefore exercise far greater caution when considering the use of AI software. Editors and journals expect reviewers to evaluate the significance of the study and manuscript. The review sheet can, and perhaps should, be simple and straightforward, written in their own English. Because confidentiality cannot be independently verified and complete deletion cannot be guaranteed, reviewers should refrain from uploading manuscripts or review sheets to AI tools.

## Regulation of Artificial Intelligence (AI) Use in Peer Review

AI, such as large language models (LLMs), for example, ChatGPT, has already been considerably used in medical writing, both in manuscript preparation and in peer review process, thus attracting the attention of both authors and reviewers. Looking through journal guidelines, use of AI tools for peer review seems to occupy less space than use of AI tools for manuscript writing ^(1), (2), (3)^.

Though there are considerable variations in journal regulations regarding use of AI tools for writing ^(4)^, there are fewer variations in those regarding use of AI tools for peer review ^(4)^. Among them, I consider, and I believe many also consider, that the most critical regulation is the prohibition against uploading the submitted manuscript or related data to AI tools or other software. I have some concerns regarding this issue. Here, I focus on use of AI tools in peer review by reviewers and will not address use of AI tools by editors, journals, or publishers. Thus, this Opinion is written from the perspective of a reviewer.

## Regulations: What Is Described

The Guidelines for Reviewers of the JMA Journal (JMAJ) are reviewer-friendly, both in content and presentation ^(1)^, and are broadly consistent with major international guidelines ^(2), (3)^. The JMAJ guideline states that “reviewers are prohibited from uploading the manuscript to software or AI-assisted tools/technologies where confidentiality is not assured.” Almost all guidelines contain similar statements ^(2), (3)^. This caution is reasonable in principle; however, AI algorithms are complex, and assurances of confidentiality cannot be independently verified by users. I believe that manuscripts under review should not be uploaded to AI tools or related software, irrespective of whether confidentiality appears to be preserved or is explicitly claimed to be preserved.

For example, some LLMs, when asked, state that the uploaded manuscript content will not be retained or that memory will be deleted after use; however, reviewers have no practical means to confirm whether such a deletion actually occurs. Similarly, even when AI tools are advertised as having no memory, operating offline, or lacking internet connectivity, such claims cannot be externally validated by individual users. From a reviewer’s standpoint, such unverifiable assurances may reasonably cause unease.

## Asking LLMs to Edit a Review Sheet: Is It Also Dangerous?

Many journals and international guidelines ^(2), (3), (4)^, including JMAJ ^(1)^, do not prohibit the use of LLMs to edit a reviewer-drafted review sheet. I have a strong concern regarding this regulation. Reviewers often write some “keywords” in the review sheet. Let us assume an extreme example. Shinya Yamanaka, a Nobel Prize winner, identified four factors that induce pluripotency: Oct4, Sox2, Klf4, and c-Myc. Let us assume this original paper has been submitted to a journal. It is highly expected that some reviewers will describe these four factors in their review sheets because they are the key points. What if reviewers ask ChatGPT to linguistically edit this review sheet? This highly confidential information is then uploaded. Will such important information not “leak” from this single act? Can we guarantee that it does not occur? I will next show a humble experiment regarding the “confidential statement” of LLMs.

## A Humble Experiment: ChatGPT! Can You Preserve 100% Confidentiality?

On February 27, 2026, I asked ChatGPT-5, “If I ask you to completely delete the uploaded data or description, will it completely disappear from the internet world? Is there a 0% possibility that some data or description remains somewhere in the system?” ChatGPT-5 answered, “*I can delete uploaded information when you request it, but even deleted items may remain within the platform’s broader data systems for service improvement purposes, so truly confidential or critical material should not be uploaded. Because zero risk is theoretically impossible in any data science or AI system, uploading confidential material inevitably carries a very small but non-zero possibility of exposure.*”

I describe this without editing because this statement is very important. Some people may misunderstand the word “memory.” LLMs can delete information from the personal “store” upon request, and thus, “no memory” is true in this sense; however, importantly, LLMs may use inputted data as training material for system improvement. In this sense, once data have been uploaded, complete deletion, meaning removing it entirely from the system, is theoretically impossible. How often and when such confidential data may appear is unknown; however, it is important to note that confidentiality cannot be fully preserved, irrespective of how rarely such appearance may occur.

## Different Stances between Authors and Reviewers

Authors’ and reviewers’ roles and situations are fundamentally different in publication ^(5)^. Regarding use of AI tools, authors may upload their own manuscripts prior to submission, for example, for language checking, under journal regulations. This may naturally increase the risk of content leakage: authors do so voluntarily and therefore accept that possibility. Reviewers, by contrast, are entrusted with unpublished work that does not belong to them. Given these differing responsibilities, reviewers should exercise far greater caution than authors when considering the use of AI software.

As stated, uploading the review sheet should, in principle, be avoided, and uploading the entire manuscript should be avoided even more to protect the confidentiality of others’ data. It is very difficult to avoid describing “core” or “keywords” in the review sheet. What if “Oct4, Sox2, Klf4, and c-Myc” were leaked before publication of the paper? Making the matter worse, who leaked the information and how it was leaked may not be determined.

## Reconsider the Fundamental: Does Peer Review Require AI?

Peer review does not inherently require the use of AI tools. The JMAJ guidelines ^(1)^ state that reviewers should accept invitations only when they have “adequate expertise to provide an authoritative assessment.” Reviewers with such expertise may not need to rely on AI tools for peer review ^(5)^. The primary role of a reviewer is to evaluate the significance and validity of the study.

A review sheet does not need to be linguistically perfect. Clear and simple comments are sufficient. “X should be Y,” “Z needs expansion,” or “W rather than V” may be enough. If a reviewer wishes to suggest a nuanced wording change but cannot clearly express it, it may be preferable to leave this to the editor or journal staff rather than uploading the review sheet to AI tools. Language polishing is secondary to scientific evaluation.

## Thus, Reviewers Should Not Upload to AI

Reviewers should not upload submitted manuscripts or review sheets to AI tools. Until confidentiality can be independently verified, such uploading should be avoided. Regardless of whether a review is detailed or brief, preserving confidentiality is a fundamental responsibility of reviewers. Clear and straightforward comments written in one’s own English are sufficient.

Peer review is essential to scientific publication. For this reason, it should remain grounded in the responsibilities entrusted to reviewers.

## Article Information

### Author Contributions

Shigeki Matsubara: Conceptualization, investigation, and manuscript writing. Shigeki Matsubara meets the ICMJE guidelines for authorship, and provided final approval for the submitted manuscript and agreed to be accountable for all aspects of the work.

### Conflicts of Interest

None

### Approval of Institutional Review Board

Not applicable.

### Patient Anonymity

Not applicable.

### Informed Consent

Not applicable.

### Declaration of AI Use

During the preparation of this work, the author (Shigeki Matsubara) asked ChatGPT-5 whether complete deletion of inputted data is impossible. The response from ChatGPT-5 is described in the manuscript. The answer was not edited and is presented as originally produced. The main manuscript was first drafted entirely by the author without AI assistance. After completion of the human-written draft, ChatGPT-5 was used solely for language editing. No conceptual arguments, interpretations, or structural components of the main manuscript were generated by AI. The author takes full responsibility for all content.

### Data availability

All data are described in the text.

### Synopsis

Reviewers should not upload the submitted manuscript and their review sheets to artificial intelligence-related tools even though these tools declare preserving confidentiality.
